# Identification and reporting of heart failure with preserved ejection fraction in Sub-Saharan Africa: a scoping review of diagnostic approaches, echocardiographic assessment, and evidence gaps

**DOI:** 10.1186/s43044-026-00779-8

**Published:** 2026-09-14

**Authors:** Faith Adedayo Adejumo, Samuel Gbolagunte Olaniyan, Temilade Patience Adejumo, Sandra Kikelomo Odunaro, Humuani Ajibola Oba, Boluwatife Juwon Falope, Olanike Elizabeth Kasali, Oluwayimika Oluwatobi Obielodan, Nicholas Aderinto

**Affiliations:** 1 Bowen University, Iwo, Nigeria; 2 Ladoke Akintola University of Technology Teaching Hospital, Ogbomoso, Nigeria; 3 University of Ilorin Teaching Hospital, Ilorin, Nigeria

**Keywords:** Heart failure with preserved ejection fraction, Sub-Saharan Africa, Echocardiography, Diastolic dysfunction, Natriuretic peptides, HFpEF phenotyping

## Abstract

**Background:**

Heart failure with preserved ejection fraction (HFpEF) is increasingly recognized as a major heart failure phenotype globally; however, its diagnosis is complex and is recommended to require integrated assessment of clinical features, echocardiographic abnormalities, and biomarkers. In Sub-Saharan Africa (SSA), where diagnostic resources are limited, HFpEF identification may rely on simplified approaches, potentially leading to under-recognition and heterogeneity in reporting. This review evaluates how HFpEF is identified and reported in SSA, with emphasis on diagnostic approaches, echocardiographic characterization, and biomarker use.

**Methods:**

A scoping review of observational studies reporting heart failure with left ventricular ejection fraction (LVEF) data in Sub-Saharan Africa (SSA) was conducted using predefined eligibility criteria and reported in accordance with PRISMA-ScR. PubMed, Google Scholar, Web of Science, and African Journals Online (AJOL) were searched for relevant studies published in English from database inception to May 2026. Data were extracted on HFpEF definitions, echocardiographic parameters, biomarker use, and diagnostic approaches and frameworks used during the respective study periods. Contemporary HFpEF diagnostic frameworks, including HFA-PEFF and H₂FPEF, were considered separately when interpreting the compatibility of reported phenotyping approaches with current diagnostic concepts.

**Results:**

10 observational studies involving 3,821 patients with heart failure, published between 2009 and 2024, were included. Studies were conducted in South Africa, Nigeria, Senegal, Côte d’Ivoire, Cameroon, and Tanzania, with two multicountry Sub-Saharan African registries also represented. HFpEF definitions were inconsistent, with most studies relying primarily on LVEF ≥ 50% without application of structured diagnostic algorithms. Only two studies reported dedicated HFpEF cohorts, while the remainder identified HFpEF as a subgroup within broader heart failure populations. Echocardiographic reporting was highly variable; left ventricular hypertrophy and left atrial enlargement were the most frequently reported structural abnormalities, but diastolic function parameters were inconsistently assessed, and formal grading was rare. Natriuretic peptides were infrequently measured and seldom integrated into diagnostic classification. No study applied contemporary multiparametric diagnostic frameworks such as HFA-PEFF or H₂FPEF scoring systems.

**Conclusion:**

HFpEF in SSA is predominantly identified using simplified ejection fraction-based criteria, with limited integration of echocardiographic diastolic assessment and biomarker data. This results in heterogeneous and potentially non-standardized phenotyping across studies, limiting comparability and accurate disease estimation. Strengthening diagnostic standardization through structured echocardiographic reporting, improved access to natriuretic peptide testing, and consideration of adapted multiparametric HFpEF algorithms may help improve diagnostic consistency and research quality in the region. These findings should be interpreted in the context of the heterogeneous and predominantly hospital-based evidence currently available from SSA.

**Supplementary Information:**

The online version contains supplementary material available at https://doi.org/10.1186/s43044-026-00779-8.

## Introduction

Heart failure (HF) is a major health challenge affecting over 55 million people worldwide [1], and its prevalence continues to rise due to population aging and increasing cardiometabolic disease [2]. In Sub-Saharan Africa, heart failure represents a significant and growing health problem with distinct epidemiological patterns, including younger age, hypertension dominance, and non-ischemic causes [3]. These epidemiological features are consistently reported across registry and observational studies in the region; however, available evidence is largely derived from hospital-based studies, and comprehensive population-level characterization of heart failure in Sub-Saharan Africa remains limited.

Within this heterogeneous heart failure population, heart failure with preserved ejection fraction (HFpEF) has emerged as a major and increasingly recognized clinical phenotype. HFpEF is a clinical syndrome characterized by signs and symptoms of heart failure in the presence of left ventricular ejection fraction (LVEF) of ≥ 50% and further supported by either elevated natriuretic peptide levels or other congestion evidence [4]. HFpEF accounts for approximately half of all heart failure cases encountered in contemporary clinical practice [4]. Unlike heart failure with reduced ejection fraction, HFpEF is characterized by complex abnormalities in ventricular relaxation, chamber compliance, and cardiovascular reserve, reflecting a distinct underlying pathophysiological profile [4]. As these abnormalities may occur despite preserved systolic function, identification of HFpEF remains considerably more challenging, as left ventricular ejection fraction alone is insufficient to confirm or exclude the diagnosis. Consequently, the diagnosis of HFpEF requires a multiparametric approach that integrates clinical assessment with echocardiographic structural and functional parameters and circulating biomarkers to improve diagnostic certainty. To standardize this diagnostic process, several standardized diagnostic guidelines have been proposed, such as the ESC heart failure recommendations, HFA-PEFF diagnostic score, and the H₂FPEF scoring system, aiming to systematize the evaluation of suspected HFpEF [5].

Echocardiography also remains central to the diagnosis of HFpEF, as it provides the structural and functional information required to establish the condition. Beyond ejection fraction, it provides information about diastolic dysfunction, LV hypertrophy, left atrial enlargement and filling pressures [6]. Importantly, no single echocardiographic parameter is sufficient for diagnosis, and they are interpreted collectively rather than in isolation; therefore, comprehensive reporting of these parameters is essential for consistent diagnosis. However, the range and completeness of echocardiographic parameters reported are highly variable across studies, reflecting a lack of standardization in the assessment of HFpEF in actual clinical practice. This variability may affect the accuracy of HFpEF identification across studies, leading to differences in reported prevalence and limiting comparability of findings.

In many Sub-Saharan settings, the diagnosis of HFpEF is constrained by limited access to comprehensive echocardiography, variable adherence to diagnostic algorithms, and inconsistent availability of natriuretic peptide testing [7]. As a result, the evaluation of HFpEF in most of these settings is often based on simplified or incomplete echocardiographic assessments, with disproportionate reliance on LVEF rather than comprehensive diastolic function evaluation. This simplified echocardiographic evaluation may potentially contribute to the misclassification or under-recognition of HFpEF within heart failure cohorts, potentially obscuring its burden [5]. In addition, studies and registries in Sub-Saharan Africa do not consistently report key HFpEF-defining echocardiographic and biomarker variables, limiting accurate phenotypic classification and cross-study comparability. Therefore, available evidence suggests that HFpEF identification in Sub-Saharan Africa appears to be heterogeneous across clinical practice and published studies. Sub-Saharan Africa was selected as the focus of this review because the region faces unique challenges in HFpEF diagnosis, including constrained access to advanced cardiac imaging, limited availability of natriuretic peptide testing, and a paucity of region-specific evidence to inform diagnostic practice.

Despite increasing recognition of HFpEF as a distinct and prevalent heart failure phenotype, there are limited studies examining diagnostic approaches, echocardiographic reporting practices, and adherence to standardized diagnostic criteria in the region. Accordingly, this review sought to answer the following question: How is HFpEF identified and reported in studies from Sub-Saharan Africa? Specifically, we evaluated diagnostic approaches, echocardiographic reporting practices, biomarker utilization, and evidence gaps in HFpEF phenotyping across the region.

## Methods

### Review design and literature search

This study was conducted as a scoping review to map how heart failure with preserved ejection fraction (HFpEF) has been defined, diagnosed, phenotyped, and reported in Sub-Saharan Africa (SSA). The review was reported in accordance with the Preferred Reporting Items for Systematic Reviews and Meta-Analyses extension for Scoping Reviews (PRISMA-ScR). The review protocol was not prospectively registered.

The review question was: How has HFpEF been defined, diagnosed, phenotyped, and reported in studies conducted in Sub-Saharan Africa? The objectives were to map the definitions and diagnostic approaches used to identify HFpEF, describe variation in echocardiographic and biomarker assessment, characterize the application of diagnostic standards and frameworks across different study periods, and identify gaps in HFpEF phenotyping and reporting across the region.

Eligibility was structured according to the Population–Concept–Context (PCC) framework. The population comprised adults aged ≥ 18 years with heart failure or heart-failure-related populations in whom left ventricular ejection fraction (LVEF) was reported. The concept was HFpEF identification and phenotyping, including definitions of preserved ejection fraction, clinical and echocardiographic diagnostic criteria, structural and functional echocardiographic parameters, biomarkers, and diagnostic guidelines or frameworks. The context was Sub-Saharan Africa, including studies conducted in individual countries or across multiple SSA countries.

A literature search was conducted in PubMed, Google Scholar, Web of Science, and African Journals Online (AJOL) from database inception to May 2026. Searches were limited to studies published in English. These databases were selected to provide coverage of biomedical, multidisciplinary, and African regional literature relevant to HFpEF in SSA. Search terms combined concepts related to heart failure, HFpEF or preserved ejection fraction, echocardiography or diastolic dysfunction, and Sub-Saharan Africa. The database-specific search strategies and search outputs are provided in Supplementary Table S1.

### Study selection

FAA and NA independently screened all retrieved records at the title and abstract stage. Records considered potentially eligible were subsequently assessed in full text by FAA and NA independently against the predefined eligibility criteria. FAA and NA independently verified eligibility at the full-text stage, and disagreements at either stage were resolved through discussion and consensus.

Duplicate records were identified and removed during the deduplication process before title and abstract screening and were therefore not considered full-text exclusions.

**Fig. 1:**
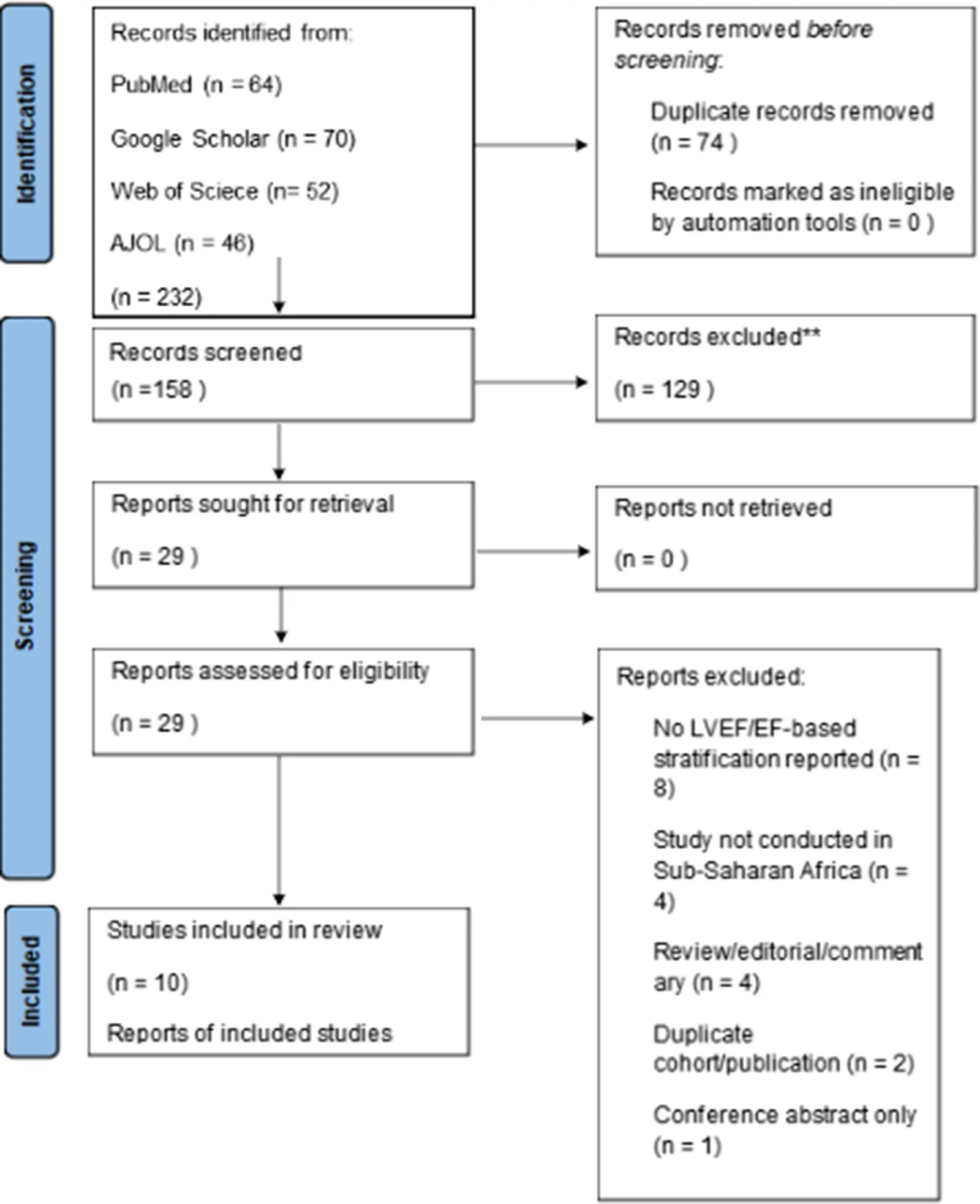
PRISMA-ScR flow diagram of the literature search and study selection process

The study-selection process was documented using a PRISMA-ScR flow diagram (Fig. 1) showing the numbers of records identified, screened, assessed for eligibility, and included, together with the reasons for full-text exclusion.

### Definition and handling of HFpEF

HFpEF definitions varied considerably across included studies, reflecting differences in diagnostic approaches and evolving guideline standards over time. For the purpose of this review, HFpEF-related data were extracted strictly as reported by the original studies.

Studies that explicitly defined HFpEF using study-specific criteria or guideline-based definitions were reported as originally described. In studies that did not explicitly define HFpEF, patients classified within preserved left ventricular ejection fraction categories (generally ≥ 50% or > 50%) were extracted as HFpEF-relevant subgroups based on study-defined stratification. The diagnostic standards and criteria used by each study were interpreted in the context of the relevant study period, and contemporary frameworks were considered separately when assessing compatibility with current HFpEF diagnostic concepts.

Where HFpEF was reported as a subgroup within a broader heart failure population, data were extracted directly from that subgroup where available. No reclassification of heart failure phenotype beyond original study definitions was performed.

### Inclusion criteria

Studies were included if they: (1) involved adult populations (≥ 18 years) with heart failure conducted in sub-Saharan Africa; (2) reported left ventricular ejection fraction (LVEF) or provided EF-based classification enabling the identification of preserved EF subgroups; and (3) were observational in design, including cohort studies, registries, or cross-sectional studies.

### Exclusion criteria

We excluded case reports, reviews, editorials, commentaries, animal studies, studies conducted outside Sub-Saharan Africa, and studies that did not report LVEF or another measure permitting EF-based classification. Studies without sufficient information to identify or characterize a preserved-EF subgroup were also excluded.

Duplicate records were removed during the deduplication process before screening and were not included among the reasons for full-text exclusion. Full-text exclusions were categorized according to the specific eligibility criterion not met, including ineligible population, ineligible context, absence of relevant LVEF/EF data, ineligible study design, or other predefined eligibility criteria.

### Data extraction

Data were extracted using a standardized framework developed for the review. FAA and NA independently extracted data from all included studies, and discrepancies were resolved through discussion and consensus.

The following information was extracted: study characteristics, including author, year, country, study design, setting, duration, and sample size; characteristics of the heart failure population; method used to identify HFpEF; LVEF threshold and other diagnostic criteria; echocardiographic parameters, including structural and diastolic measures; biomarkers, particularly BNP or NT-proBNP; diagnostic standards or frameworks used during the relevant study period; and other features relevant to HFpEF phenotyping and reporting.

### Data synthesis

Extracted data were synthesized descriptively, narratively, and thematically to map patterns in HFpEF identification and reporting across the included studies. Findings were organized into tables comparing study characteristics, HFpEF definitions, echocardiographic assessment, biomarker use, diagnostic approaches, and overall phenotyping practices.

The synthesis distinguished between the diagnostic approaches used by studies during their respective study periods and their compatibility with contemporary concepts of HFpEF diagnosis. No quantitative meta-analysis was undertaken because the primary objective was to map diagnostic and reporting practices and evidence gaps rather than estimate a pooled treatment effect or formally assess intervention effectiveness.

A formal risk-of-bias or methodological quality assessment was not performed, as the primary purpose of this scoping review was to map the available evidence and characterize variation in HFpEF identification, phenotyping, and reporting rather than to estimate intervention effects or determine certainty of evidence.

## Results

### Characteristics of included studies

Ten observational studies reporting HFpEF-related data in Sub-Saharan Africa were included [8–17]. Studies were conducted in South Africa, Senegal, Côte d’Ivoire, Nigeria, Cameroon, and Tanzania, with two multicountry registries involving up to eight Sub-Saharan African countries. Most studies were hospital-based and undertaken in tertiary or academic referral centers.

Study designs included prospective cohort studies (*n* = 5), cross-sectional studies (*n* = 3), retrospective studies (*n* = 1), and one prospective descriptive study. Study duration ranged from 4 months to 5 years among studies reporting this information; one study reported no study duration. Across all included studies, a total of 3,821 patients with heart failure were evaluated, with cohort sizes ranging from 32 to 1,294 participants.

Only two studies (20%) enrolled dedicated HFpEF cohorts [8, 9], whereas the remaining eight studies (80%) identified HFpEF as a subgroup within broader heart failure populations [10–17]. Among studies reporting subgroup data, 759 patients were classified as HFpEF. Female predominance was reported in five studies, male predominance in two studies, one study reported an equal sex distribution, and two studies did not report sex distribution. Mean age ranged from 48.2 to 72 years. Study characteristics are summarized in Table 1.

**Table 1:** Characteristics of studies reporting HFpEF-related data in Sub-Saharan Africa

S/*N*	Author and Year	Country	Study design	Setting	Study duration	HF Population Type	Total HF Cohort (*n*)	HFpEF Patients (*n*)	Sex distribution	Mean age (years)
1	Van Hoogland-van Heerden et al. (2024)	South Africa	Cross-sectional	Academic Hospital	14 months	Dedicated HFpEF cohort	66	66	Female predominance	54
2	Mboup et al. (2013)	Senegal	Prospective descriptive study	Tertiary Hospital	12 months	Dedicated HFpEF cohort	32	32	Female predominance	65
3	Traore et al. (2017)	Côte d’Ivoire	Retrospective study	Tertiary cardiac Hospital	12 months	Mixed Heart Failure Cohort	257	64	Male predominance	57.3
4	Karaye KM et al. (2021)	Multiple Sub-Saharan African countries (Nigeria, South Africa, Sudan, Uganda, Mozambique)	Prospective study	Tertiary Hospital	12 months	Mixed Heart Failure Cohort	1,294	207	Male predominance	59
5	Damasceno A et al. (2012)	Multiple Sub-Saharan African countries (Nigeria, South Africa, Sudan, Uganda, Mozambique, Kenya, Senegal, Ethiopia)	Prospective study	Tertiary Hospitals	36 months	Mixed Heart Failure Cohort	1,006	NR	Equal	52.3
6	Karaye et al. (2013)	Nigeria	Prospective observational comparative hospital study	Tertiary Hospital	12 months	Mixed Heart Failure Cohort	100	32	Female predominance	48.2
7	Boombhi et al. (2020)	Cameroon	Analytical cross-sectional study	Tertiary Hospitals	4 months	Mixed Heart Failure Cohort	189	86	NR	65.9
8	Maro et al. (2009)	Tanzania	Prospective study	Tertiary Hospital	5 years	Mixed Heart failure Cohort	360	122	Female predominance	72
9	Ojji et al. (2009)	Nigeria	Prospective observational study	Tertiary Hospital	28 months	Mixed HF Cohort	340	80	NR	NR
10	Adebayo et al. (2009)	Nigeria	Cross-sectional	Tertiary Hospital	NR	Mixed HF Cohort	177	70	Female predominance	52

Footnote Two of the ten included studies (20%) were dedicated HFpEF cohorts; the remaining studies reported HFpEF as a subgroup within broader heart failure populations

### Diagnostic approaches used to identify HFpEF

HFpEF identification was predominantly based on preserved left ventricular ejection fraction (LVEF). Six studies used an EF threshold of ≥ 50% [8, 9, 11, 13, 16, 17], and four used a threshold of > 50% [10, 12, 14, 15].

Although all included studies identified HFpEF on the basis of preserved LVEF, they differed substantially in the rigor of HFpEF ascertainment. Based on the comprehensiveness of the diagnostic approach, the studies can be broadly classified into two groups. The first comprised studies with comprehensive HFpEF ascertainment, represented by Van Hoogland-van Heerden et al. [8] and Mboup et al. [9], which enrolled predefined HFpEF cohorts and incorporated dedicated clinical and echocardiographic evaluation beyond preserved LVEF. The second comprised studies with limited HFpEF ascertainment [10–17], in which HFpEF was identified retrospectively as an LVEF-defined subgroup within a broader heart failure cohort.

Echocardiographic completeness varied considerably across the included studies. Structural or functional echocardiographic criteria beyond preserved LVEF were incorporated into HFpEF assessment in four studies [8, 9, 14, 17], including left ventricular hypertrophy, left atrial enlargement, and Doppler-derived indices of diastolic function. Explicit demonstration of diastolic dysfunction formed part of the diagnostic definition in three studies [9, 10, 14]. In contrast, the remaining studies relied largely on preserved LVEF with limited reporting of structural or diastolic parameters, reflecting substantial variability in the comprehensiveness of echocardiographic evaluation.

Biomarker integration was uncommon, with natriuretic peptide testing reported in only a few studies and incorporated into HFpEF diagnostic classification in only one study [10].

The included studies spanned different diagnostic eras. Diagnostic criteria were therefore interpreted in relation to the standards applicable during the respective study periods. Earlier studies generally used EF-based classification and echocardiographic criteria that reflected the diagnostic approaches available at the time, whereas more recent studies incorporated more comprehensive structural, functional, or biomarker-based assessment. No included study explicitly applied the HFA–PEFF algorithm or H₂FPEF score. Contemporary frameworks were therefore considered separately when assessing the compatibility of the reported diagnostic approaches with current HFpEF diagnostic concepts, rather than being used to retrospectively reclassify participants.

These findings are summarized in Table 2.

**Table 2:** Comparative summary of HFpEF diagnostic definitions, echocardiographic parameters, biomarker use, and guideline frameworks in included studies

S/*N*	Author (Year)	HFpEF Definition Used	EF Threshold	Diastolic Function Criteria Reported	Echocardiographic Parameters Reported	Biomarkers Used	Diagnostic Framework / Guideline
1	Van Hoogland-van Heerden et al. (2024)	Clinical HFpEF diagnosis based on ESC 2016 guidelines; symptoms/signs of HF with echocardiographic evidence of preserved EF and structural heart disease (LVH and/or LA enlargement)	≥ 50%	E/A ratio, E/e′ (septal), e′ and a′ velocities; no formal diastolic dysfunction grading reported	LVEF, LVEDd, LVESd, IVSd, LVPWd, LVPWs, LVM, LVMI, RWT, LA diameter, Ao diameter, LA/Ao, LA/LV, E, A, E/A, e′, a′, E/e′	NT-proBNP, Galectin-3	ESC 2016 heart failure guidelines (no formal HFpEF scoring system used)
2	Mboup et al. (2013)	HFpEF defined as HF with preserved EF plus echocardiographic evidence of isolated LV diastolic dysfunction	≥ 50%	Em/Ea ratio > 15; Em/Am, pulmonary venous flow (Ap–Am), deceleration time; diastolic dysfunction categorized (impaired relaxation, pseudonormal, restrictive)	LVEF, LVEDd, LVESd, LV mass index, LA size/area, mitral inflow (Em, Am), tissue Doppler Ea, Em/Ea, deceleration time, pulmonary artery pressure	NR	ESC diastolic dysfunction recommendations (implied), no formal HFpEF scoring system
3	Traore et al. (2017)	HFpEF defined as HF symptoms/signs + NT-proBNP elevation + LVEF > 50% + LA dilatation + diastolic dysfunction (E/E′ >13)	> 50%	E/E′ ratio > 13 used as primary diastolic dysfunction criterion; LA enlargement included in definition; no formal grading of diastolic dysfunction reported	LVEF, LA dilation (volume-based), E/E′ ratio; limited full diastolic parameter set reported	NT-proBNP	Local institutional criteria based on echocardiography + NT-proBNP (ESC-aligned but not fully standardized HFpEF scoring system
4	Karaye KM et al. (2021)	HF classified by symptoms/signs of HF with EF-based phenotyping; HFpEF defined as part of EF stratification (no dedicated HFpEF diagnostic definition applied beyond EF category)	≥ 50%	NR	Only LVEF reported	NR	Boston criteria for HF diagnosis + ESC EF-based classification
5	Damasceno A et al. (2012)	NR	> 50%	mitral inflow and deceleration time	LVEF, LV dimensions, LA size, wall thickness, mitral inflow velocities,	NR	Clinical + echocardiography-based AHF diagnosis per ESC-style criteria
6	Karaye et al. (2013)	Defined as symptomatic HF with LVEF ≥ 50%	≥ 50%	MV E: A ratio, LA size, and Doppler indices	LVEF, LVEDD, LVESD, IVSd, posterior wall thickness, LA diameter, MV E/A ratio, mPAP; routine transthoracic echo parameters	NT-proBNP reported (log10 NT-BNP provided) but not used as diagnostic threshold-based criterion	NR
7	Boombhi et al. (2020)	Symptoms/signs of HF + LVEF > 50% + structural heart disease (LVH and/or LA dilation) and/or diastolic dysfunction	> 50%	E/e’ ≥13 and/or e’ <9 cm/s for diastolic dysfunction	LV mass index, LVEDD/LVESD, LA size, RA size, SPAP, LVH pattern, mitral flow profiles, EF (Teichholz), LV geometry	NR	ESC 2016 clinical HF definition + echo criteria (LVEF + structural + diastolic indices)
8	Maro et al. (2009)	NR	> 50%	NR	LVEF (M-mode/2D), LV dimensions, left atrial size, wall motion assessment, Doppler indices (mitral inflow E/A ratio, deceleration time), LV hypertrophy, valvular assessment	NR	Clinical diagnosis of CHF using modified Framingham criteria + echocardiographic confirmation of systolic function classification
9	Ojji et al. (2009)	Clinical HF with LVEF ≥ 50%.	≥ 50%	Deceleration time (DT), impaired filling pattern, pseudonormal filling, restrictive filling	LVEF, LV dimensions, interventricular septal thickness, posterior wall thickness	NR	HFpEF classified solely by EF ≥ 50%
10	Adebayo et al. (2009)	Symptomatic HF diagnosed by Framingham criteria + preserved EF subgroup (HFNEF) within HF cohort	≥ 50%	Mitral inflow Doppler: E, A, E/A ratio, deceleration time (DT); no formal diastolic grading system used	LVEF (Teichholz), LVEDD, LVESD, IVSd, PWTd, RWT, LV mass, LA size, aortic root, mitral inflow (E/A, DT), fractional shortening	NR	Framingham clinical HF criteria + echocardiography-based EF stratification

Footnote The HFpEF population type (dedicated HFpEF cohort or HFpEF subgroup within a mixed heart failure cohort) for each study is presented in Table 1

### Echocardiographic reporting practices

LVEF was reported in all included studies (10/10) [8–17] and represented the only universally documented echocardiographic parameter.

Left atrial size or enlargement was reported in nine studies [8–11, 13–17], while left ventricular hypertrophy, wall thickness, or LV mass indices were reported in eight studies [8–11, 13–16].

Assessment of diastolic function was reported in eight studies [8–10, 13–17], most commonly using mitral inflow E/A ratio, deceleration time, and/or tissue Doppler indices. However, reporting was inconsistent across cohorts.

Tissue Doppler-derived parameters (e′, a′, or E/e′) were reported in four studies [8, 10, 13, 14]. Only one study formally classified diastolic dysfunction into impaired relaxation, pseudonormal, and restrictive filling patterns [9].

Overall, echocardiographic characterization ranged from comprehensive multiparametric assessment [8, 9] to EF-only classification [11, 16], limiting comparability of HFpEF phenotyping across studies.

### Biomarker utilization

Natriuretic peptide measurements were reported in a small number of studies [8, 10, 13]. However, among the included studies, only one study incorporated natriuretic peptide elevation as a component of HFpEF diagnostic classification [10]. This is notable given the established diagnostic role of natriuretic peptides in HFpEF algorithms and ESC recommendations [6].

### Cross-study synthesis of reporting completeness

Across studies, HFpEF phenotyping was predominantly EF-based rather than integrated structural–functional–biomarker-based assessment. No study applied full HFA-PEFF or H₂FPEF diagnostic frameworks.

Overall, substantial heterogeneity in diagnostic definitions, echocardiographic reporting, and biomarker use was observed (Table 3).

**Table 3:** Synthesis of methodological and reporting patterns in HFpEF identification across included studies

S/*N*	Domain	Pattern observed across studies
1	HFpEF identification approach	Predominantly EF-based classification within general heart failure cohorts; only a minority of studies used explicitly defined HFpEF cohorts, resulting in heterogeneous case ascertainment and limited diagnostic specificity.
2	Use of formal HFpEF diagnostic frameworks	No study applied contemporary HFpEF scoring systems such as HFA-PEFF or H₂FPEF; most relied on EF cut-offs (≥ 50%) combined with variable clinical or echocardiographic criteria, reflecting absence of standardized diagnostic adjudication.
3	Structural echocardiographic phenotyping (LV/LA geometry)	Reporting was inconsistent across studies; while LV hypertrophy and left atrial enlargement were commonly assessed, the depth of structural quantification (e.g., LV mass index, relative wall thickness, volumetric LA measures) varied substantially between studies.
4	Diastolic function assessment	Largely underreported or inconsistently applied; only a subset of studies reported Doppler-derived indices (e.g., E/e′, E/A ratio), and formal grading of diastolic dysfunction using guideline-based algorithms was rare
5	Biomarker integration	Very limited incorporation of natriuretic peptides (BNP/NT-proBNP), with only isolated studies reporting biomarker data; biomarkers were rarely used as diagnostic or stratification criteria for HFpEF phenotyping.
6	HFpEF phenotyping depth	Overall phenotyping was superficial in most studies, with reliance on EF stratification alone rather than integrated structural–functional–biomarker profiling; comprehensive HFpEF characterization was uncommon.
7	Study design limitations	Majority were hospital-based observational cohorts or registries, limiting generalizability; few prospective HFpEF-focused studies were available, and most analyses were secondary within general HF populations.
8	Temporal limitations	Several studies predated current HFpEF guideline frameworks (ESC 2016 and later HFA recommendations), contributing to variability in diagnostic criteria and incomplete phenotyping.
9	Geographic and institutional representation	Evidence is unevenly distributed across Sub-Saharan Africa, with a concentration in tertiary or academic referral centres, limiting representation of community-level HFpEF burden.
10	Overall evidence summary	The body of evidence is fragmented, heterogeneous, and non-standardized, with significant variability in diagnostic criteria, echocardiographic reporting, and biomarker use, resulting in limited comparability across studies and weak HFpEF-specific inference.

## Discussion

### Principal findings

HFpEF has been increasingly reported in studies from Sub-Saharan Africa; however, its identification and characterization within the published literature remain inconsistent and incompletely standardized. Although all included studies reported HFpEF-related data, substantial heterogeneity was observed in diagnostic definitions, echocardiographic assessment, and biomarker utilization. Most studies classified HFpEF primarily using preserved LVEF thresholds, while only a minority incorporated comprehensive structural or functional echocardiographic criteria. Furthermore, natriuretic peptide testing was infrequently reported, and contemporary diagnostic algorithms were absent across all included studies. In this review, studies were considered dedicated HFpEF cohorts when HFpEF represented the primary population of interest and participants were enrolled specifically based on HFpEF-related criteria. In contrast, studies were classified as reporting HFpEF subgroups when preserved EF patients were identified within broader heart failure cohorts. This distinction was based on the study objectives, inclusion criteria, and reported methodology of the original publications.

The most important finding is that HFpEF phenotyping in the region remains largely EF-based rather than multiparametric. While preserved ejection fraction is an essential component of diagnosis, contemporary evidence recognizes HFpEF as a complex syndrome requiring integration of symptoms, biomarkers, structural cardiac abnormalities, and objective evidence of elevated filling pressures rather than reliance on LVEF alone [18–20]. The predominance of simplified EF-based classification observed in this review therefore raises concerns regarding diagnostic precision and comparability across studies.

A second important finding was the marked variability in echocardiographic reporting. Although left ventricular hypertrophy and left atrial enlargement were frequently documented, detailed characterization of diastolic dysfunction was inconsistently reported. This limits confidence in the diagnostic validity of HFpEF classification and suggests that reported HFpEF cohorts across the region may represent heterogeneous populations identified using substantially different criteria.

### Diagnostic phenotyping gaps relative to contemporary HFpEF standards

Over the past decade, HFpEF diagnosis has evolved considerably. In addition to the development of structured diagnostic pathways like the HFA-PEFF algorithm and H₂FPEF score, emphasis has also been placed on appropriate phenotyping [4, 21, 22]. This is because symptoms and preserved EF alone lack specificity. Against this contemporary framework, the studies included in this review demonstrate a substantial implementation gap. No study applied either the HFA-PEFF or H₂FPEF diagnostic framework, and several studies identified HFpEF largely through EF stratification within broader heart failure cohorts. Even among studies reporting structural abnormalities, the depth of phenotyping varied considerably, with inconsistent reporting of tissue Doppler indices, filling pressure estimates, left atrial volume measurements, and formal grading of diastolic dysfunction.

Beyond differences in diagnostic criteria, limited phenotypic characterization also restricts the ability to distinguish the diverse disease substrates contributing to HFpEF presentations in Sub-Saharan Africa. HFpEF represents a heterogeneous clinical syndrome rather than a single disease entity, and preserved ejection fraction with diastolic dysfunction may occur in the context of multiple underlying cardiac conditions [4, 18]. In the SSA setting, hypertensive heart disease and diabetes-related myocardial dysfunction are likely important contributors, reflecting the substantial burden of hypertension, diabetes, and other cardiometabolic risk factors across the region [23, 24]. However, other conditions, including endomyocardial fibrosis, chronic pericardial disease, and infiltrative cardiomyopathies such as cardiac amyloidosis, may also present with preserved ejection fraction and impaired diastolic function [25–27]. These conditions may produce distinct patterns and severity of diastolic abnormalities through differences in myocardial compliance, ventricular restriction, and filling pressures [25–27]. However, the limited use of comprehensive echocardiographic assessment, biomarker evaluation, and advanced imaging in available studies prevents reliable differentiation of these underlying phenotypes. Consequently, some patients classified as HFpEF in SSA studies may represent heterogeneous preserved EF heart failure populations with differing pathophysiological mechanisms, highlighting the need for improved phenotypic characterization in future research.

Importantly, these findings should not necessarily be interpreted as methodological shortcomings of individual investigators. Several studies were published before the introduction of contemporary HFpEF diagnostic frameworks such as the HFA–PEFF algorithm and H₂FPEF score [9, 10, 12, 13, 15–17], and many originated from healthcare environments where advanced echocardiographic modalities and biomarker testing may not be routinely available. This temporal variation in the evidence base should be considered when interpreting differences in HFpEF characterization, as diagnostic approaches have evolved substantially over time. Nevertheless, the cumulative effect is a fragmented evidence base in which diagnostic heterogeneity limits cross-study comparability and complicates interpretation of HFpEF prevalence and clinical characteristics across the region.

### Echocardiographic reporting and the challenge of phenotypic characterization

LVEF was universally reported, reflecting widespread availability of conventional echocardiographic assessment. However, reporting of parameters required for comprehensive HFpEF characterization was considerably less consistent. Structural abnormalities, particularly left ventricular hypertrophy and left atrial enlargement, represented the most frequently reported markers of cardiac remodeling. In contrast, measures of diastolic function such as E/e′ ratio, tissue Doppler velocities, and formal diastolic dysfunction grading were inconsistently documented. Furthermore, the criteria used to define abnormal filling pressures were not uniform across studies, with variation in reported E/e′ thresholds and tissue Doppler criteria (i.e., E/e′ >13 in some studies versus combined criteria such as E/e′ ≥13 and/or reduced e′ velocity in others). This variability further limits interpretation of the overall frequency of reported diastolic dysfunction and complicates cross-study comparison. This discrepancy may reflect that, within the included studies, structural assessment was more commonly reported than detailed functional evaluation.

The limited reporting of diastolic indices has important implications. Diastolic dysfunction represents a defining pathophysiological feature of HFpEF and contributes substantially to elevated filling pressures and exercise intolerance [28, 29]. Inadequate characterization of diastolic function may therefore contribute to diagnostic uncertainty and obscure important phenotypic differences between patient populations. Consequently, future studies should prioritize standardized reporting of key echocardiographic parameters to improve diagnostic reproducibility and facilitate meaningful comparisons across cohorts.

### Limited biomarker integration in HFpEF identification

One of the most striking findings of this review was the limited incorporation of natriuretic peptides into HFpEF assessment. Only a small number of studies reported BNP or NT-proBNP measurements, and biomarker results were seldom integrated into diagnostic classification. This observation is particularly important because natriuretic peptides occupy a central position within contemporary HFpEF diagnostic pathways and are recommended by major international guidelines as objective markers supporting the diagnosis of heart failure [5, 6, 30, 31]. Beyond diagnosis, natriuretic peptides provide important prognostic information and contribute to risk stratification across the spectrum of heart failure phenotypes [6].

The low frequency of biomarker utilization observed in this review likely reflects resource limitations, cost considerations, and restricted laboratory availability in many healthcare settings across Sub-Saharan Africa. However, reduced access to natriuretic peptide testing may also contribute to under-recognition of HFpEF and limit diagnostic confidence in patients with equivocal echocardiographic findings. Expanding access to biomarker testing may therefore represent an important step toward improving HFpEF characterization within the region.

### Phenotypic interpretation of HFpEF in Sub-Saharan Africa

An important implication of this review is that HFpEF, as reported in Sub-Saharan African studies, may not represent a uniformly defined clinical entity consistent with contemporary guideline definitions. Instead, the current evidence suggests that HFpEF is variably identified using predominantly left ventricular ejection fraction–based criteria, with limited incorporation of systematic diastolic function assessment and natriuretic peptide testing. As a result, the populations labelled as HFpEF across studies likely reflect a heterogeneous group of patients with preserved systolic function rather than a consistently defined phenotype.

An additional consideration is the potential overlap between HFpEF and other heart failure phenotypes, particularly HF with mildly reduced ejection fraction (HFmrEF). Since most included studies were not dedicated HFpEF cohorts and several identified HFpEF within broader heart failure populations, variation in LVEF thresholds and classification approaches may have resulted in the inclusion of patients with intermediate or mixed phenotypes. This further limits direct comparison across studies and highlights the need for dedicated HFpEF cohorts using contemporary EF categories and multiparametric diagnostic criteria.

The frequent reporting of structural abnormalities such as left ventricular hypertrophy and left atrial enlargement suggests that many of these cases are driven by chronic pressure overload states, particularly hypertension-related cardiac remodeling. However, in the absence of comprehensive functional and biomarker evaluation, it remains difficult to confirm whether these cases meet contemporary diagnostic thresholds for HFpEF or represent earlier or broader stages of structural heart disease. This interpretation is particularly relevant in the SSA context, where hypertension represents a dominant and increasingly recognized contributor to the heart failure burden and is strongly associated with hypertensive cardiac remodeling.

However, the characterization of left atrial remodeling was limited, as none of the included studies reported indexed left atrial volume (LAVI), with most relying on linear left atrial measurements or non-indexed measures of atrial size. This represents an important limitation in HFpEF phenotyping, as LAVI is incorporated within contemporary diagnostic frameworks, including the HFA-PEFF algorithm [32], as a marker of structural cardiac remodeling and chronically elevated filling pressures.

### Implications for research and clinical practice

The findings of this review have important implications. From an epidemiological perspective, variability in HFpEF definitions and incomplete phenotyping likely contributes to inconsistent prevalence estimates across SSA studies. Differences in reported HFpEF burden may therefore reflect methodological variation as much as true geographic or population-level differences. From a research perspective, the absence of standardized diagnostic approaches limits comparability across studies and complicates interpretation of regional HFpEF epidemiology.

The interpretation of these findings should also consider the broader epidemiological context of heart failure in sub-Saharan Africa, where hypertension represents a major contributor to heart failure burden. The frequent identification of structural abnormalities such as left ventricular hypertrophy and left atrial enlargement across included studies may reflect the influence of chronic pressure overload and hypertension-related cardiac remodeling. Improving HFpEF phenotyping in this setting is therefore particularly important to distinguish true HFpEF from other manifestations of hypertensive heart disease and to better characterize the spectrum of preserved ejection fraction heart failure in the region. Potential publication bias should also be considered, as studies with more comprehensive HFpEF characterization or stronger methodological approaches may be more likely to be published, while less extensively phenotyped cohorts may be underrepresented in the available literature.

Clinically, incomplete phenotyping may contribute to misclassification of patients and hinder identification of clinically meaningful HFpEF subgroups. As therapeutic options for HFpEF continue to expand, accurate diagnosis and phenotypic characterization will become increasingly important for ensuring appropriate patient management.

Available evidence also suggests that HFpEF in Sub-Saharan Africa should not be regarded as a benign condition. In high-income populations, long-term registry data have demonstrated 5-year mortality comparable to that of HFrEF, while the KaRen cohort reported mortality increasing from approximately 15% at one year to over 70% at ten years [4, 33]. Longitudinal studies have shown that approximately 39% of patients with HFpEF experience deterioration in left ventricular ejection fraction over time, including 21% progressing to HFmrEF and 18% progressing to HFrEF [34]. Although evidence from Sub-Saharan Africa remains limited, a post hoc analysis of the THESUS-HF registry involving 888 Black African patients demonstrated no significant differences in 60-day death or readmission or 180-day mortality between patients with HFpEF, HFmrEF, and HFrEF [35], suggesting that HFpEF is not a comparatively benign phenotype in African populations. However, prospective longitudinal data from the region are lacking, and it therefore remains uncertain whether progression from HFpEF to HFrEF follows a similar trajectory in African populations. These findings further emphasize that improving diagnostic standardization and phenotypic characterization will be essential not only for accurate case identification but also for reliable prognostic assessment, risk stratification, and the development of context-specific management strategies in Sub-Saharan Africa.

### Toward a pragmatic diagnostic approach for HFpEF in Sub-Saharan Africa

Current guideline-recommended diagnostic pathways for HFpEF remain difficult to implement consistently across many healthcare settings in Sub-Saharan Africa owing to limited access to natriuretic peptide testing, comprehensive echocardiographic assessment, advanced cardiac imaging, and specialist expertise. Consequently, HFpEF diagnosis has frequently relied on preserved left ventricular ejection fraction alone, resulting in considerable heterogeneity in case ascertainment and limited phenotypic characterization.

**Fig. 2:**
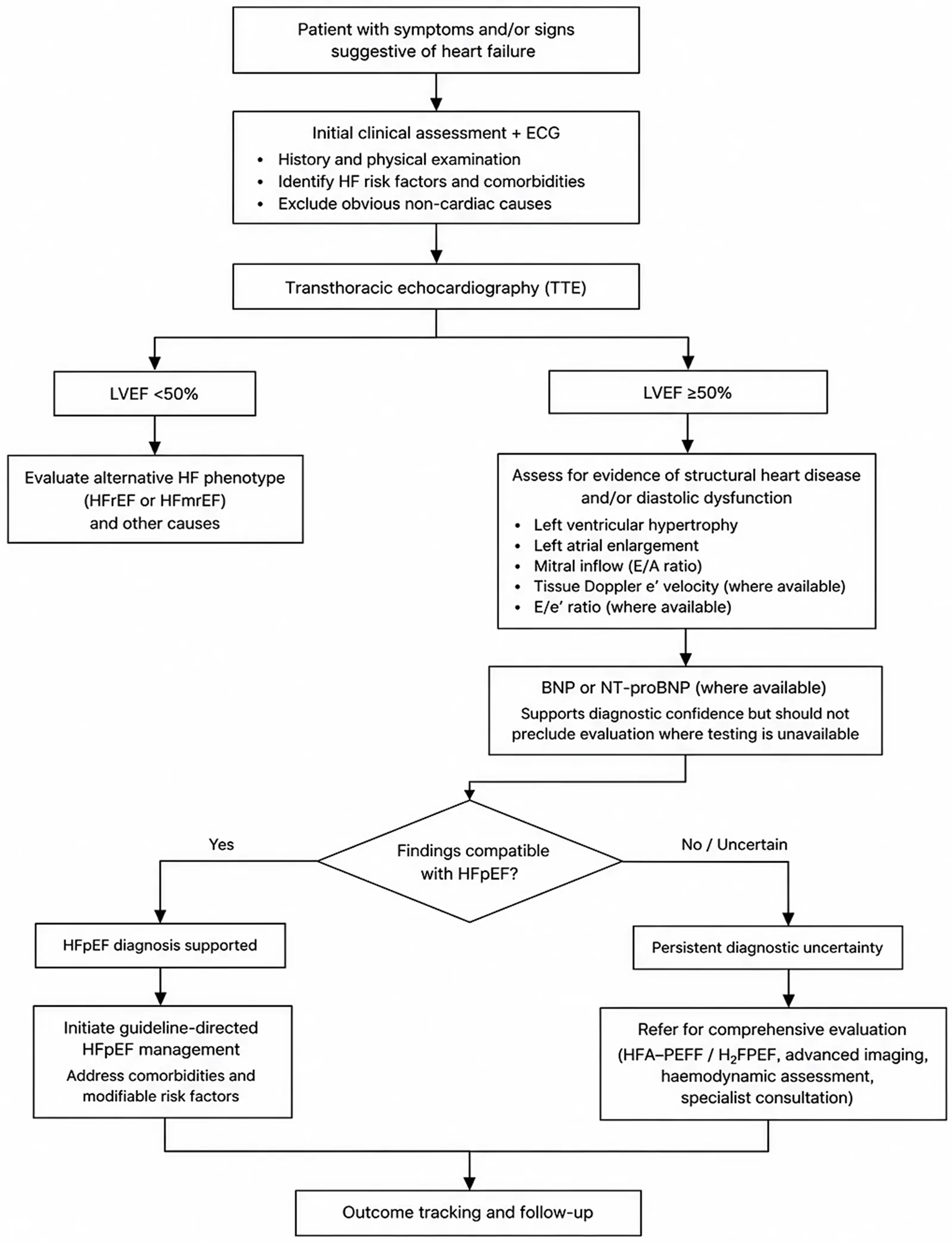
Conceptual framework for a pragmatic approach to HFpEF evaluation in Sub-Saharan Africa

Based on the evidence synthesized in this review, we present a conceptual framework for a pragmatic approach to HFpEF evaluation in Sub-Saharan Africa (Fig. 2). Rather than replacing established diagnostic frameworks such as the HFA–PEFF algorithm or the H₂FPEF score, this framework is intended to support more consistent clinical evaluation where access to comprehensive diagnostic resources is limited. As illustrated in Fig. 2, evaluation should begin with careful clinical assessment and electrocardiography, followed by transthoracic echocardiography to confirm preserved left ventricular ejection fraction and assess for structural cardiac abnormalities and evidence of diastolic dysfunction. Natriuretic peptide testing should be incorporated whenever available to strengthen diagnostic confidence but should not preclude further evaluation in patients with a high clinical suspicion of HFpEF where biomarker testing is unavailable. Patients with persistent diagnostic uncertainty should, whenever feasible, be referred for comprehensive assessment using validated multiparametric diagnostic algorithms, advanced cardiac imaging, or specialist evaluation.

Importantly, this proposed framework represents a pragmatic, stepwise approach rather than a new diagnostic algorithm. As diagnostic capacity continues to improve across Sub-Saharan Africa, progressive implementation of contemporary guideline-directed diagnostic pathways should remain the long-term objective. Until then, adoption of this pragmatic framework may improve diagnostic consistency, facilitate more accurate phenotypic characterization, and enhance comparability across future clinical studies conducted within the region.

### Future directions

Future research in Sub-Saharan Africa should move beyond descriptive reporting of HFpEF and prioritize standardized, diagnostically rigorous phenotyping approaches that align with contemporary international guidelines but remain feasible within resource-limited settings.

First, there is a need to implement standardized HFpEF diagnostic frameworks, such as simplified adaptations of the HFA-PEFF algorithm, that incorporate minimum essential criteria, including structural echocardiographic markers (left atrial volume and left ventricular mass index), objective evidence of diastolic dysfunction (E/e′ ratio and tissue Doppler indices), and, where feasible, natriuretic peptide measurement. This would improve diagnostic consistency and reduce reliance on isolated ejection fraction–based classification. This recommendation directly addresses the predominant EF-based classification, absence of formal HFpEF diagnostic frameworks, and limited phenotyping depth identified in Table 3.

In addition, future studies should prioritize the establishment of prospective, HFpEF-specific cohorts and multicenter registries across Sub-Saharan Africa using harmonized definitions. This recommendation is informed by the evidence gaps identified in this review, particularly the predominance of hospital-based mixed heart failure cohorts, limited availability of HFpEF-specific studies, and lack of standardized phenotyping approaches (Table 3). Such studies would allow more accurate estimation of disease prevalence, facilitate phenotype stratification, and enable meaningful comparison with global HFpEF populations.

There is also a need for capacity strengthening in echocardiographic assessment, particularly in the routine acquisition and reporting of diastolic function parameters and left atrial volumetric indices. Standardized reporting templates could significantly improve reproducibility and diagnostic quality. This recommendation is based on the inconsistent reporting of structural parameters and limited assessment of diastolic function identified in Table 3.

Finally, future research should explore context-adapted biomarker strategies, including scalable access to natriuretic peptide testing, to support diagnostic accuracy in resource-constrained environments. This addresses the limited incorporation of natriuretic peptides and biomarker-based phenotyping observed across included studies (Table 3). Without integration of biomarkers and diastolic assessment, HFpEF identification in the region will remain incomplete and potentially misleading [5, 31].

## Strengths and limitations of the study

This scoping review provides a broad mapping of HFpEF identification and reporting practices across Sub-Saharan Africa. It describes variation in diagnostic approaches, echocardiographic assessment, and biomarker use, while identifying important gaps in HFpEF phenotyping and reporting. By synthesizing evidence from multiple countries and study periods, the review provides an overview of how HFpEF has been characterized across the region and highlights areas requiring further research and improved standardization.

Regarding limitations, the included studies were heterogeneous in design, sample size, and reporting quality. Most were hospital-based, limiting generalizability to community populations. Additionally, the geographic distribution of included studies was uneven, with evidence concentrated in a limited number of Sub-Saharan African countries, which may restrict the applicability of findings to regions with different healthcare systems, disease patterns, and diagnostic resources. Variability in echocardiographic reporting limited direct comparability across studies. Additionally, reliance on secondary extraction may have resulted in incomplete capture of HFpEF-specific variables due to inconsistent reporting in primary studies. A formal risk-of-bias or methodological quality assessment was not performed, which limits the ability to systematically evaluate the strength of evidence from included studies. Furthermore, the heterogeneity in HFpEF definitions, study populations, and reported outcomes precluded meta-analysis and generation of pooled estimates. The search strategy was also limited by the restriction to English-language publications and the exclusion of grey literature, conference abstracts, and unpublished data, which may have resulted in the omission of relevant evidence.

Furthermore, the predominance of tertiary hospital-based cohorts introduces potential referral and selection bias, as these settings may preferentially capture patients with more advanced disease or greater access to specialized cardiovascular assessment. In addition, publication bias cannot be excluded, as studies from academic referral centers with greater diagnostic capacity may be more likely to be represented in the published literature.

## Conclusion

In the published literature from Sub-Saharan Africa, heart failure with preserved ejection fraction has been identified predominantly through preserved ejection fraction-based classification, with variable incorporation of additional structural, functional, and biomarker-based criteria. Across published studies, substantial heterogeneity exists in HFpEF definitions and reporting practices, particularly regarding diastolic function assessment and natriuretic peptide utilization. This variability highlights the need for cautious interpretation of existing HFpEF data from the region and underscores the importance of standardized phenotyping approaches to improve consistency in future studies.

This variability also means that it cannot be determined whether patients classified as HFpEF in the region would satisfy contemporary guideline-based diagnostic criteria, as individual patient-level data were not available for retrospective application of these criteria. This limits comparability with global datasets and may contribute to uncertainty in understanding the true epidemiology of HFpEF in Sub-Saharan Africa.

To address these gaps, the findings of this review suggest that future efforts should consider the implementation of standardized, multiparametric diagnostic approaches that integrate structural, functional, and biochemical assessment. Improving echocardiographic reporting practices, expanding access to natriuretic peptide testing, and developing prospective HFpEF registries may help improve diagnostic accuracy, enhance research quality, and support evidence-based understanding and management of heart failure in the region.

## Electronic supplementary material

Below is the link to the electronic supplementary material.

Supplementary Material 1

## Data Availability

No datasets were generated or analysed during the current study.

## Abbreviations

HF: Heart Failure
HFpEF: Heart Failure with Preserved Ejection Fraction
LVEF: Left Ventricular Ejection Fraction
EF: Ejection Fraction
SSA: Sub–Saharan Africa
ESC: European Society of Cardiology
HFA: PEFF–Heart Failure Association Pre–test Assessment, Echocardiography and Natriuretic Peptide Score, Functional Testing, Final Etiology
H₂FPEF: Heavy, Hypertensive, Atrial Fibrillation, Pulmonary Hypertension, Elder, Filling Pressure Score
LV: Left Ventricle / Left Ventricular
LVH: Left Ventricular Hypertrophy
LA: Left Atrium / Left Atrial
LVEDD: Left Ventricular End–Diastolic Diameter
LVESD: Left Ventricular End–Systolic Diameter
LVEDd: Left Ventricular End–Diastolic Dimension
LVESd: Left Ventricular End–Systolic Dimension
LVMI: Left Ventricular Mass Index
LVM: Left Ventricular Mass
IVSd: Interventricular Septal Thickness in Diastole
PWTd: Posterior Wall Thickness in Diastole
LVPWd: Left Ventricular Posterior Wall Thickness in Diastole
LVPWs: Left Ventricular Posterior Wall Thickness in Systole
RWT: Relative Wall Thickness
LA/Ao: Left Atrial–to–Aortic Root Ratio
LA/LV: Left Atrial–to–Left Ventricular Ratio
BNP: B–type Natriuretic Peptide
NT: proBNP–N–terminal Pro–B–type Natriuretic Peptide
mPAP: Mean Pulmonary Artery Pressure
SPAP: Systolic Pulmonary Artery Pressure
E/A: Early–to–Late Mitral Inflow Velocity Ratio
E′ (e′): Early Diastolic Mitral Annular Velocity
A′ (a′): Late Diastolic Mitral Annular Velocity
E/e′: Ratio of Early Mitral Inflow Velocity to Early Diastolic Mitral Annular Velocity
DT: Deceleration Time
RA: Right Atrium
CHF: Congestive Heart Failure
HFNEF: Heart Failure with Normal Ejection Fraction
Ao: Aortic Root
HFmrEF: Heart Failure with Mildly Reduced Ejection Fraction
