# Supplementary material for: Identification and reporting of heart failure with preserved ejection fraction in Sub-Saharan Africa: a scoping review of diagnostic approaches, echocardiographic assessment, and evidence gaps
Source: Egypt Heart J. 2026 Sep 14;78:66. doi: 10.1186/s43044-026-00779-8 (PMC13574737; doi:10.1186/s43044-026-00779-8)
Supplement: Supplementary file 1 [file 43044_2026_779_MOESM1_ESM.docx]

**Supplementary Table S1. Electronic databases searched, search strategies, and search outputs**

| **Database** | **Search date/coverage** | **Search strategy** | **Search output**  **(records retrieved)** |
| --- | --- | --- | --- |
| PubMed | From database inception to May 2026 | ("heart failure" OR "congestive heart failure" OR HF) AND ("heart failure with preserved ejection fraction" OR HFpEF OR "heart failure with normal ejection fraction" OR HFNEF OR "preserved ejection fraction") AND (echocardiograph* OR "diastolic dysfunction" OR "diastolic function" OR "left ventricular ejection fraction" OR LVEF) AND ("Sub-Saharan Africa" OR Africa) | 64 |
| Web of Science | From database inception to May 2026 | TS=("heart failure" OR "congestive heart failure" OR HF) AND TS=("heart failure with preserved ejection fraction" OR HFpEF OR "heart failure with normal ejection fraction" OR HFNEF OR "preserved ejection fraction") AND TS=(echocardiograph* OR "diastolic dysfunction" OR "diastolic function" OR "left ventricular ejection fraction" OR LVEF) AND TS=("Sub-Saharan Africa" OR Africa) | 52 |
| Google Scholar | From database inception to May 2026 | A broad combination of the following concepts was used: "heart failure" AND ("preserved ejection fraction" OR HFpEF OR "normal ejection fraction") AND (echocardiography OR "diastolic dysfunction" OR LVEF) AND ("Sub-Saharan Africa" OR Africa). | 70 |
| AJOL | From database inception to May 2026 | ("heart failure" OR HF) AND ("preserved ejection fraction" OR HFpEF OR "normal ejection fraction" OR HFNEF) AND (echocardiography OR "diastolic dysfunction" OR LVEF OR "ejection fraction") AND (Africa OR "Sub-Saharan Africa") | 46 |

**Note:** TS = Topic; searches terms in the Web of Science Topic fields (Title, Abstract, Author Keywords, and Keywords Plus).
